# Cortical functional activity in patients with generalized anxiety disorder

**DOI:** 10.1186/s12888-016-0917-3

**Published:** 2016-07-07

**Authors:** Yiming Wang, Fangxian Chai, Hongming Zhang, Xingde Liu, Pingxia Xie, Lei Zheng, Lixia Yang, Lingjiang Li, Deyu Fang

**Affiliations:** Department of Psychiatry, Affiliated Hospital of Guizhou Medical University, Guiyang, Guizhou 550004 China; Department of Cardiolog, The General Hospital of Jinan Military Region, Jinan, 250031 China; Department of Cardiolog, Affiliated Hospital of Guizhou Medical University, Guiyang, Guizhou 550004 China; The second Xiangya Hospital, Central South University, 139# Renmin road, Changsha, Hunan 410011 China; Department of Pathology, Northwestern University Feinberg School of Medicine, Chicago, 60611 USA

**Keywords:** Psychiatry, Generalized anxiety disorder, EEG nonlinear analysis, Correlation dimension, Cortical functional activity

## Abstract

**Background:**

The neurological correlates of Generalised Anxiety Disorder (GAD) are not well known, however there is evidence of cortical dysregulation in patients with GAD. The aim of the study was to examine cortical functional activity in different cerebral regions in patients with GAD using electroencephalogram (EEG) nonlinear analysis to evaluate its contribution of anxiety severity.

**Methods:**

The cohorts consisted of 64 patients diagnosed with GAD as classified by the Structured Clinical Interview for the Diagnostic and Statistical Manual of the American Psychiatric Association-IV-TR. Anxiety severity was assessed using the Hamilton Rating Scale for Anxiety (HAMA) severity score, with 7 ≤ scores ≤ 17 indicating mild anxiety as A group (*n* = 31) and 18 and above indicating moderate-severe anxiety as B group (*n* = 33). Participants with clinical levels of depression symptoms were excluded. A healthy control group comprising 30 participants was matched for age and gender. Closed eyes EEGs were conducted, and between-group differences on non-linear parameter Correlation Dimension (D2) were analyzed. The association of D2 value with HAMA scores was analyzed using multiple linear stepwise regression.

**Results:**

Compared with the control group, D2 values were increased in anxiety groups (*P* < .05). For those with mild anxiety, this difference occurred in the left prefrontal regions (*P* < .05). For those with moderate-severe anxiety, significantly greater D2 values were observed in all of the cerebral regions, especially in the left cerebral regions and right temporal lobe (*P* < .01). When compared with those with mild anxiety, D2 values were significantly greater for those with moderate-severe anxiety in the right temporal lobe and all left cerebral regions except for left occipital lobe (*P* < .05). A positive correlation was observed between D2 values and moderate-severe anxiety HAMA scores.

**Conclusions:**

The increased D2 values were found in the majority of cerebral regions in GAD patients, especially in the left cerebral regions and the right temporal lobe. The increased GAD severity positively correlates to the D2 values in a larger number of cerebral regions. This analysis method can potentially be used as a complementary tool to examine dysfunctional cortical activity in GAD.

## Background

General anxiety disorder (GAD) is a common and chronic anxiety disorder characterized by persistent worry [1]. While advancements have been made in our understanding of the psycho-physiology of GAD in recent years, the neurological correlates of GAD remain poorly understood. Previous studies using functional magnetic resonance imaging (fMRI) have demonstrated that the anterior cingulate and the medial prefrontal regions were activated in GAD patients [2], suggesting some cortical activity was dysfunctional in GAD patients [3]. Other fMRI studies found that both the ventral prefrontal cortex (vPFC) and amygdala region are correlation with social anxiety disorder [4]. What remains unclear however, is whether other cerebral regions are also differentially engaged in people with anxiety when compared to those without, and whether there is a relationship between severity of anxiety disorder and cerebral region functional activity.

EEG is an important noninvasive method to explore cortical neuronal activity by placing electrodes on the scalp, including waveform investigations and power spectra analysis [5–8]. Molina et al [9] reported that patients with obsessive compulsive disorder (OCD) have increased beta and theta power in the EEG and perfusion in frontal regions. However, the complex dynamic changes in brain cannot be discriminated using the technique [10]. Furthermore, EEG with nonlinear features and complexity parameter, for example correlation dimension (D2), approximate entropy (ApEn), fractal dimension (FD), have been suggested as more suitable techniques for analyzing cerebral dynamic activity. The linear EEG is sufficient and well used for the clinical symptom classification of brain diseases as well as brain electronic signal alterations under different stress conditions, but the non-linear EEG analysis in fractal dimension [11] provides a better analysis of quantification to identify EEG changes in response to special stimulating conditions such as the GAD. D2 is known as one of nonlinear parameter for studying dynamic behaviors.

Another study using quantitative electroencephalography (qEEG) in patients with anxiety have shown decreased alpha, beta and theta activity, especially in cerebral middle and central regions [12], and basal instability in cortical arousal has also been found [13]. Nonlinear EEG analysis is a new research tool that is being utilized in the study of different physiologic conditions, for example sleep and dementia [14, 15]. The D2 in the EEGs nonlinear analysis, is defined as an index of complexity of information processing representing the cerebral cortical dynamics and behavior. D2 has been used in experimental and clinical settings in studying schizophrenia [16]. Decreased D2 values have been detected in patients with seizure epilepsy [17], Alzheimer’s disease [18], schizophrenia [19], and depression [20].

The aim of this study was to use EEG nonlinear dynamics analysis to compare functional changes of the cerebral cortex in patients with or without GAD and to evaluate the impact of the severity of anxiety on cortical functional activity.

## Methods

### Subjects

The study sample consisted of 64 patients with GAD (30 men and 34 women, aged 20–63 years) recruited from the department of psychiatry at the Affiliated Hospital of Guiyang Medical University, from June 2010 to January 2011. All consenting participants met diagnostic criteria for GAD according to the Structured Clinical Interview for the Diagnostic and Statistical Manual of Mental Disorders-IV-TR. (DSM-IV) and scored ≥7 on the Hamilton Anxiety Rating Scale (HAMA) [21]. Participants with clinical levels of depression symptoms (defined by a score of ≥ 53 on the Zung Self-Rating Depression Scale (SDS) [22]) were excluded. Participants were categorised according to their HAMA scores as having either “mild anxiety” (7 ≤ scores ≤ 17) or “moderate-severe anxiety” (HAMA ≥ 18) for evaluating the impact of the severity of anxiety on cortical functional activity. Healthy control participants (*n* = 30) who did not meet the diagnostic criteria for GAD according to the Structured Clinical Interview for the Diagnostic and Statistical Manual of Mental Disorders-IV-TR (DSM-IV) and scored ≤ 6 on the Hamilton Anxiety Rating Scale (HAMA) were recruited. Diagnostic interviews were conducted by two psychiatrists. Exclusion criteria for all participants included secondary somatic diseases (hyperthyroidism, hypertension and coronary heart disease).

Selective serotonin-reuptake inhibitors (SSRIs) were routinely used to treat patients with anxiety, concomitant short term use of benzodiazepines was allowed (alprazolam (Xanax), 0.8 mg/day, bid for 2 weeks Yabao pharmaceutical Taiyuan Pharmaceutical Co., Ltd.).

## Assessment

### The severity of anxiety

Severity of anxiety symptoms was assessed using the HAMA. This is a 14 item clinician rated scale, with each item rated 0-4 where a higher score indicates more severe symptoms [23], with a total anxiety score range from 0 to 56, score ≤ 17 indicates mild severity, where ≥ 18 shows mild to moderate, and 25–30 indicates moderate-severe.

### EEG nonlinear analysis

EEG was conducted in a quiet room with soft lighting. EEG electrodes were placed in accordance with international standards for leads 10–20, specifically left and right forehead (FP1, FP2); left and right frontal (F3, F4); left and right central (C3, C4); left and right parietal (P3, P4); left and right occipital (O1, O2); left and right anterior temporal (F7, F8); left and right temporal (T3, T4); left and right posterior temporal (T5, T6). The left and right earlobe (A1, A2) were used as reference sites. The data was acquired and analyzed using 16-channel EEG (Wireless Digital EEG, ZN16E, Chengdu, China), the EEG with international 10–20 system was used to obtain EEGs in the subjects [24].

In resting state, EEG signals were recorded from each participant, who was asked to close eyes and keep on a relax condition without movement during the 5 min data collection period. EEG data in each interval (180 s) were selected from the 5 min collection period and divided into 6 segments with 30s each for nonlinear processing. Any artifact sources caused by eye-blink and movement in EEG recording were eliminated by the component analysis system based on their different frequencies and patterns as this analysis system is able to recognize the unique patterns of electronic signaling caused by the eye blink.

The D2 value was calculated using a nonlinear software packet embedded in the 16-channel Wireless Digital EEG system. D2 is an index of the variation within a selected time series, reflecting the complexity of a non-linear system. Nonlinear EEG analyzes the brain activity using nonlinear dynamic mathematics and chaos theory, which allows us to monitor the dynamics and complicated electronic activity [25]. Natural and biological phenomena often displays features of nonlinear dynamics, particularly respond to perturbing stimuli or initial conditions [26].

The D2 values are shown as the average of data collected from all the brain regions of each patients as indicated in Table 2. The differences among controls and patients with anxiety of mild symptoms, or moderate-severe symptoms were statistically evaluated based on the method of Lacasa [27].

### Statistics analyses

Data analysis was carried out using SPSS 19.0 software. The results were described using Median ± Quartile (M ± Q). The Rank Sum Test was carried out among the three groups (control, mild anxiety, moderate-severe anxiety). *P* < 0.05 was considered as statistical significant. The final regression model was Ŷ = 2.7985-0.417X1 + 0.533 *X*2 (Ŷ: estimates the value of the correlation dimension D2, X1: the patient’s age, *X*2: HAMA score of patients). The association between D2 and HAMA scores was analyzed between moderate-severe anxiety (B group) and control groups using multiple linear stepwise regression, significance was set at α = .05.

## Results

### Demographic characteristics

The key characteristics of the patient cohorts are displayed in Table 1. No significant differences were found between the control, GAD A and B groups in the demographic variables (age, sex) between the control and GAD A and B groups. Their smoking status and blood pressure ratings of patients and control groups were indicated, the percentages of smoking populations and average blood pressure in each group are comparable (Table 1).

**Table 1:** Demographic data between the control and GAD groups (Mean ± SEM)

Items	Control (*n* = 30)	A (*n* = 31)	B (*n* = 33)
Ages (years)	45.41 ± 6.33	46.41 ± 7.56	46.37 ± 6.42
(Ranges)	20–65	20–63	21–58
Gender (male/female) (n)	14/16 (47 %/53 %)	15/16 (48 %/52 %)	15/18 (46 %/54 %)
Resting SBP (mm Hg)	120 ± 20	125 ± 22	130 ± 20
Resting DBP (mm Hg)	70 ± 12	72 ± 10	76 ± 10
Smokers/non-smokers (n)	15/15 (50 %/50 %)	16/15 (52 %/48 %)	18/15 (55 %/45 %)

No significant differences in the demographic variables (age, sex, blood pressure, smoking) between the control and GAD A and B groups, were observed

*SBP* systolic blood pressure, *DBP* diastolic blood pressure, *GAD* Generalised Anxiety Disorder, Group A: mild symptoms of anxiety; Group B: moderate-severe symptoms of anxiety

### The comparison of Correlation Dimension between the groups

Compared with the control group, D2 values were increased in anxiety groups (*P* < .05). For those with mild anxiety, this difference occurred only in the left prefrontal regions (*P* < .05). For those with moderate-severe anxiety, significantly greater D2 values were observed in all of the cerebral regions, especially in the left cerebral regions and right temporal lobe (*P* < .01). When compared with those with mild anxiety, D2 values were significantly greater for those with moderate-severe anxiety in the right temporal lobe and all left cerebral regions except for left occipital lobe (*P* < .05) (Tables 2 and 3).

**Table 2:** The comparison of Correlation Dimension in different groups (M ± Q)

Group	n	Left prefrontal (FP1-A1)	Right forehead (FP2-A2)	Left frontal (F3-A1)	Right frontal (F4-A2)	Left central (C3-A1)	Right central (C4-A2)	Left parietal (P3-A1)	Right parietal (P4-A2)
Control	30	3.57 ± 0.48	3.53 ± 0.58	3.64 ± 0.36	3.61 ± 0.43	3.66 ± 0.44	3.66 ± 0.51	3.65 ± 0.34	3.59 ± 0.49
A	31	3.36 ± 0.24^*^	3.41 ± 0.46	3.52 ± 0.35	3.58 ± 0.37	3.55 ± 0.36	3.66 ± 0.33	3.59 ± 0.39	3.58 ± 0.37
B	33	3.66 ± 0.69^▲▲^	3.52 ± 0.59^▲^	3.95 ± 0.33^**▲▲^	3.81 ± 0.35^▲▲^	3.94 ± 0.33^**▲▲^	3.85 ± 0.27^▲▲^	3.97 ± 0.27^**▲▲^	3.80 ± 0.41^▲▲^
P		0.000	0.016	0.000	0.004	0.000	0.003	0.000	0.006

Compared with control group, ^*^ *P* < .05, ^**^ *P* < .01;compared with group A, ^▲^ *P* < .05,^▲▲^ *P* < .01

Group A: mild symptoms of anxiety; Group B: moderate to severe symptoms of anxiety

**Table 3:** The comparison of Correlation Dimension in different groups (M ± Q)

Group	n	Left occipital (O1-A1)	Right occipital (O2-A2)	Left anterior temporal (F7-A1)	Right anterior temporal (F8-A2)	Left temporal (T3-A1)	Right temporal (T4-A2)	Left posterior temporal (T5-A1)	Right posterior temporal (T6-A2)
Control	30	3.62 ± 0.59	3.69 ± 0.57	3.63 ± 0.54	3.71 ± 0.56	3.91 ± 0.69	3.86 ± 0.50	3.78 ± 0.36	3.60 ± 0.59
A	31	3.45 ± 0.61	3.42 ± 0.47^*^	3.43 ± 0.58	3.70 ± 0.46	3.66 ± 0.72	3.65 ± 0.71	3.61 ± 0.54	3.55 ± 0.54
B	33	3.99 ± 0.23^**▲▲^	3.81 ± 0.51^▲▲^	4.05 ± 0.82^**▲▲^	3.89 ± 0.36^▲▲^	4.29 ± 0.49^**▲▲^	4.01 ± 0.51^▲▲^	4.14 ± 0.51^**▲▲^	3.81 ± 0.32^▲▲^
P		0.000	0.000	0.000	0.004	0.000	0.005	0.000	0.006

Compared with control group, *P < .05, **P < .01;compared with group A, ^▲^ *P* < .05,^▲▲^ *P* < .01

Group A: mild symptoms of anxiety; Group B: moderate to severe symptoms of anxiety

### Association between D2 value and HAMA scores

**Fig. 1:**
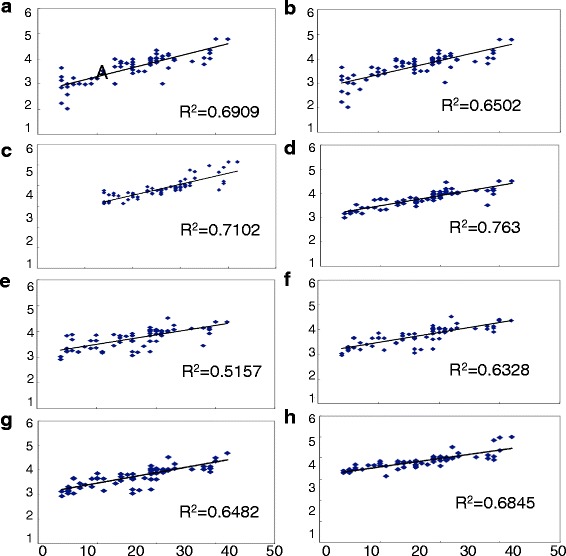
Correlation Dimension correlation with HAMA score. The Correlation Dimension values and moderate-severe anxiety HAMA scores were positively correlated (*r*_*s*_ > .63, *P* < .05) in the left prefrontal, frontal, central, parietal occipital, anterior temporal, temporal, posterior temporal regions (**a**-**h**)

Multiple linear stepwise regression analysis revealed that, for the GAD B group, D2 values and HAMA scores were positively correlated (*r* > .63, *P* < .05) in the left prefrontal, frontal, central, parietal occipital, anterior temporal, temporal, posterior temporal regions (Fig. 1).

## Discussion

This study found that individuals with GAD have dysfunctional cortical activity in the majority of cerebral regions, especially in the left cerebral regions and right temporal lobe. Moreover, more severe anxiety was associated with involvement of a larger number of cerebral regions. Specifically, we demonstrated increased values of D2 in frontal temporal regions in GAD patients, suggestive of temporal lobe cortical dysfunction. These association is unlikely affected by their smoking status and blood pressure because the percentages of smoking populations and average blood pressure in each group are comparable. Glosser et al. found that patients with anterior temporal lobectomy often develop mood disorders and the severity of psychiatric symptoms peak in the 6 months after surgery [28]. It is possible that dysfunction of the frontal and temporal regions can result in emotional changes [29], and the results of an fMRI study on patients with GAD also revealed dysfunction in the bilateral superior temporal gyrus and dorsal prefrontal cortex [30]. However, given the cross-sectional nature of these data and the multiple comparisons used, identifying the true direction of this relationship requires further investigation.

In the current report, we found increased D2 values in patients with greater GAD severity, lateralized to the left hemisphere (the dominant hemisphere as all participants were right-handed). To our knowledge, this is the first study to demonstrate significant differences in cerebral hemisphere in left and right laterality, however the machanism is not clear. Kalisch et al. [31] used the magnetic resonance imaging at 7 T to measure hippocampal volumes in a rat model of extremes in trait anxiety (experiment 1) and in a Wistar population with normal anxiety-related behavior (experiment 2). While experiment 2 yielded a strong evidence for a negative relationship which was specific for trait anxiety, results from experiment 1 globally supported the hypothesis of a positive relationship between hippocampus volume and trait anxiety. Therefore, as what the authors stated, the relationship between hippocampal volume and anxiety may be more complex than expected, thus we speculate that this increase in D2 value is associated to the cortical dysfunctional activity in these patients.

We agree with the reviewer that the temporal and hippocampal regions are not the same. It has been reported [32] that the volume in the whole-brain gray matter, not only in the hippocampal region, but also including superior temporal gyrus and midbrain, is reduced in patients with GAD. It has also been shown [33] that the reduced white matter (WM) volume were associated with dysfunctional cognitive and emotional in GAD, WM volume is correlation with symptom severity. Implying that dysfunctional activities can occur both in cerebral cortex and hippocampal regions correlation with clinical characteristics. However, it would be technically challenging to detect the hippocampal dysfunctional activation by EEG in human. Although the use of Nonlinear EEG to analyze generalized anxiety disorder has not been reported, this analysis method is suggested in further research the ralationship between cerebral cortex volume and generalized anxiety disorder, and as a complementary tool to detect dysfunctional cortical activity in GAD.

A strength of this study was the use of neuroimaging to investigate cortical dysfunctional activity. The nonlinear analysis method was sufficiently sensitive to detect differences in cortical activity between patients with different levels of anxiety, a finding that may have implications for the clinical diagnosis of GAD. Currently, a diagnosis of GAD relies on clinical judgment, using information gathered via symptom assessment and responses to standardized scales. However, a disadvantage of clinical judgment is subjectivity; diagnosis will be influenced by clinical training, experience and other subjective factors and may differ amongst clinicians. This technique, however, remains the gold standard for psychiatric diagnostic assessment.

Neuroimaging techniques may impact upon the nosology of mental disorders in the future and have the capacity to influence psychiatric practice by potentially improving clinical treatment [34]. Indeed, these have been valuable in other areas of psychiatry, for example, in depressed populations, via fMRI [35], positron emission tomography (PET) [36] and single-photon emission computed tomography (SPECT) [37, 38]. These differing neuroimaging methodologies, fMRI, PET, SPECT and EEGs nonlinear analysis offer complimentary insights into neurobiology, and each have their strengths and weaknesses. fMRI and PET techniques can not directly detect neuronal activity, neither can EEG. Linear analysis methods have limits in stability and sensitivity in detecting complicated cortex function as EEG signals originate in a highly nonlinear system [39, 40]. Further, nonlinear dynamics analysis can provide information about the neural network [41] and track the changes cerebral functional activity [42, 43] which cannot be detected with linear analysis. This technique has also shown to be more sensitive than linear analysis in detecting subtle aspects of emotional processing. Indeed, EEG nonlinear analysis has been widely used in other populations (epilepsy [44, 45], schizophrenia [46], dementia [47]), to monitor the depth of anesthesia [48–50], and is effective in evaluating patients being treated for acute carbon monoxide poisoning [51]. As the electrical activity of the human brain self-organizing nonlinear dynamic systems is complex, and the neural circuits are extensive and full of synaptic connections [52]. The non-linear method in the current study has a great potential in delineating the complexity of EEG in patients with different psychological disorders.

However, we acknowledge that there are certain limitations when interpreting these data. The sample size was not extensive, limiting ability to further analysis the relationship between anxiety disorder severity and EEG non-linear parameters. However, given the cross-sectional nature of these data and the multiple comparisons used, identifying the true direction of this relationship requires further investigation.”

## Conclusions

Patients with GAD demonstrate dysfunctional cortical activity in the majority of cerebral regions, the increased D2 values were found in the majority of cerebral regions in GAD patients, especially in all the left cerebral regions and right temporal lobe, GAD severity positively correlate with increased D2 values in a larger number of cerebral regions. Our study demonstrates that EEG dynamic non-linear analysis is appropriate for the investigation of the neurological correlates of GAD, and holds potential for the further evaluation of cortical dysfunctional activity in patients with varying levels of GAD severity and assessment of treatment effects, this analysis method is also suggested as a complementary tool to detect dysfunctional cortical activity in GAD.

## Abbreviations

D2, Correlation Dimension; DBP, diastolic bood pressure; DSM-IV, Diagnostic and Statistical Manual of Mental Disorders-IV-TR; EEG, electroencephalogram; fMRI: functional magnetic resonance imaging; GAD, Generalised Anxiety Disorder; HAMA, Hamilton Rating Scale for Anxiety; PET, positron emission tomography; SBP, systolic blood pressure; SDS, Zung Self-Rating Depression Scale; SPECT, single-photon emission computed tomography; vPFC, ventral prefrontal cortex
